# Nicotinic acid changes rumen fermentation and apparent nutrient digestibility by regulating rumen microbiota in Xiangzhong black cattle

**DOI:** 10.5713/ab.23.0149

**Published:** 2023-10-31

**Authors:** Zhuqing Yang, Linbin Bao, Wanming Song, Xianghui Zhao, Huan Liang, Mingjin Yu, Mingren Qu

**Affiliations:** 1 College of Animal Science and Technology, Jiangxi Agricultural University, Nanchang 330045, China; 2 Animal Husbandry and Veterinary Bureau of Guangchang County, Fuzhou, Jiangxi, 344900, China; 3 Jiangxi Provincial Key Laboratory for Animal Nutrition/Engineering Research Center of Feed Development, Jiangxi Agricultural University, Nanchang, 330045, China

**Keywords:** Apparent Digestibility, Microbial Composition, Nicotinic Acid, Rumen Fermentation Parameters, Xiangzhong Black Cattle

## Abstract

**Objective:**

The aim of this study was to investigate the impact of dietary nicotinic acid (NA) on apparent nutrient digestibility, rumen fermentation, and rumen microbiota in uncastrated Xiangzhong black cattle.

**Methods:**

Twenty-one uncastrated Xiangzhong black cattle (385.08±15.20 kg) aged 1.5 years were randomly assigned to the control group (CL, 0 mg/kg NA in concentrate diet), NA1 group (800 mg/kg NA in concentrate diet) and NA2 group (1,200 mg/kg NA in concentrate diet). All animals were fed a 60% concentrate diet and 40% dried rice straw for a 120-day feeding experiment.

**Results:**

Supplemental NA not only enhanced the apparent nutrient digestibility of acid detergent fiber (p<0.01), but also elevated the rumen acetate and total volatile fatty acid concentrations (p<0.05). 16S rRNA gene sequencing analysis of rumen microbiota revealed that dietary NA changed the diversity of rumen microbiota (p<0.05) and the abundance of bacterial taxa in the rumen. The relative abundances of eight Erysipelotrichales taxa, five Ruminococcaceae taxa, and five Sphaerochaetales taxa were decreased by dietary NA (p< 0.05). However, the relative abundances of two taxa belonging to *Roseburia faecis* were increased by supplemental 800 mg/kg NA, and the abundances of seven *Prevotella* taxa, three Paraprevotellaceae taxa, three Bifidobacteriaceae taxa, and two operational taxonomic units annotated to *Fibrobacter succinogenes* were increased by 1,200 mg/kg NA in diets. Furthermore, the correlation analysis found significant correlations between the concentrations of volatile fatty acids in the rumen and the abundances of bacterial taxa, especially *Prevotella*.

**Conclusion:**

The results from this study suggest that dietary NA plays an important role in regulating apparent digestibility of acid detergent fiber, acetate, total volatile fatty acid concentrations, and the composition of rumen microbiota.

## INTRODUCTION

Nicotinic acid (NA), a member of vitamin B, plays a crucial role as an essential nutrient in the energy metabolism of beef cattle [1]. Serving as a direct precursor of Coenzyme I (NAD+/NADH) and Coenzyme II (NADP+/NADPH), it actively participates in lipid metabolism, glycolysis, tissue oxidation, and respiratory function [2]. NA also participates in the conversion of ruminant volatile fatty acids (VFAs), such as acetic acid, to long-chain fatty acids [2]. It was previously thought that adult ruminants could obtain sufficient NA through microbial synthesis in the rumen or tryptophan conversion [2]. However, numerous recent studies have demonstrated that endogenous synthesis of NA is insufficient to meet the nutritional requirements of high-yielding dairy cows and intensive production of beef cattle [3–7]. NA plays a crucial role in promoting rumen microbial growth, maintaining the stable rumen microecology, and reducing the lactic acid accumulation in the rumen wall [3,7,8]. Therefore, dietary NA supplementation can improve the performance and adaptability of high-yield ruminants to diets with a high forage-to-concentrate ratio [3–7]. A high-concentrate diet supplemented with 800 mg/kg NA has the potential to alleviate subacute rumen acidosis (SARA) and stabilize rumen pH by inhibiting the proliferation of *Streptococcus bovis* or by increasing NAD^+^ production to inhibit lactate dehydrogenase activity [3,7]. Further study has revealed that NA inhibits starch utilization and stimulates fiber degradation primarily by reducing the relative abundance of *Proteobacteria*, *Succiniclasticum*, *Acetivibrio*, and *Treponema*, or increasing the abundance of *Prevotella* [3]. Additionally, supplemental NA can increase the abundance of total rumen protozoa [8–10]. However, the NA requirement of ruminants varies depending on animal species, the type of concentrate, and the roughage fed to animals [1].

The substantial secretion of testosterone from the testis of uncastrated bulls contributes to their rapid growth rate, the high proportion of lean meat in carcasses, low fat content, and enhanced feed efficiency [11]. Uncastrated bulls are preferred to be used for fattening in beef production [12], due to their higher level of serum testosterone, resulting in faster growth performance and alterations in lipid metabolism and fatty acid profiles, as demonstrated by a recent study on yellow cattle [11]. The NA induced an increase in body weight gain, which was attributed to its ruminal and metabolic effects as well as its ability to enhance feed intake in bulls [1].

Our previous study suggested that dietary NA significantly regulated the lipid and glucose metabolisms of uncastrated Xiangzhong cattle [13]. The VFAs produced by rumen fermentation play an important role in host lipid and glucose metabolism. Considering the important roles of NA in promoting rumen microbial growth and fiber degradation, we hypothesized that NA could modulate host lipid and glucose metabolisms by regulating bacterial composition and rumen fermentation to produce VFAs in uncastrated bulls. Therefore, this study compared apparent digestibility of nutrients, rumen fermentation parameters, and the composition of rumen bacteria among three treatment groups with dietary supplementation of NA at different levels.

## MATERIALS AND METHODS

### Animal care

All animal works were approved by the Animal Care and Use Committee of Jiangxi Agricultural University (No. JXAU 2011-006).

### Animals and sample collection

The experimental cattle used in this study were sourced from a Chinese indigenous breed named Xiangzhong black cattle. Twenty-one uncastrated Xiangzhong black cattle (initial body weight of 385.08±15.20 kg) at the age of 1.5 years were randomly allocated into three groups with seven individuals per group. Each group was assigned to one of the following dietary treatments: i) basal diet+0 mg NA/kg concentrate (CL), ii) basal diet + 800 mg NA /kg concentrate (NA1), iii) basal diet+1,200 mg NA/kg concentrate (NA2). The cattle were housed in individual stalls (1.7×3.5 m) and fed a diet consisting of 60% concentrate and 40% rice straw twice daily at 07:00 and 16:00, respectively, adopting *ad libitum* feeding manner. Water was provided *ad libitum*. All cattle were in good health and had not received any antibiotic treatment for two months before slaughter. The composition and nutrient levels of the basal concentrate diet are presented in Table 1. NA was purchased from Jiangsu Nantong Biotechnology Co., Ltd. (Purity >97%; China). For NA treatments, the mineral premix was initially blended with NA and subsequently added to the concentrate. After a formal experiment period of 120 days, all animals were slaughtered following the Chinese standard procedures at a commercial slaughterhouse. Rumen samples were collected from multiple sites of the rumen, equally mixed, and strained with cheese cloth to obtain rumen fluid that are used for analyses. Three out of seven rumen samples from each group were randomly chosen for 16S rRNA gene sequencing. All samples for 16S rRNA gene sequencing were promptly dipped in liquid nitrogen and transferred to a −80°C refrigerator until utilization.

### DNA extraction and 16S rRNA gene sequencing

Microbial DNA samples were extracted from rumen samples within one month after sample collection using the QIAamp Fast DNA Stool Mini Kit (Qiagen, Hilden, North Rhine-Westphalia, Germany) according to the manufacturer’s instructions. The concentration and purity of DNA samples were determined using Nanodrop-1000 and 1% agarose gel electrophoresis. The V4 region of bacterial 16S rRNA gene was amplified using the fusion primers 515F (5′-GTGCCAG CMGCCGCGGTAA-3′) and 806R (5′-GGACTACHVGGG TWTCTAAT-3′) [14]. Subsequently, the polymerase chain reaction products were normalized to equimolar quantities and subjected to sequencing on an Illumina MiSeq platform (Illumina, San Diego, CA, USA) using a pair-end 250 bp strategy.

### Bioinformatics analysis of 16S rRNA gene sequencing data

Raw sequencing data were filtered to exclude the adaptor and low-quality sequences to obtain clean reads. Clean sequence reads with overlapped fragments longer than 10 bp and zero mismatches were assembled into tags by using the FLASH program (v.1.2.11) [15]. The USEARCH software (v.7.0.1090) [16] was employed for the operational taxonomic unit (OTU) clustering at the threshold of 97% sequence identity. Alpha-diversity metrics including observed species, Shannon, Simpson, Chao, Ace, and Good’s coverage were calculated using Mothur (v.1.39.5) [17]. The beta-diversity analysis of rumen microbiota including principal component analysis (PCA), principal co-ordinates analysis (PCoA), non-metric multidimensional scaling analysis (NMDS) and analysis of similarities (ANOSIM) were conducted using QIIME (v1.80) [18]. The figures were plotted with ggplot2 in the package (v 3.5.2). The potential functional capacity of the rumen microbial community was predicted using PICRUSt 2 based on 16S rRNA marker gene sequences. Normalization, metagenome prediction and function categorization based on Kyoto encyclopedia of genes and genomes (KEGG) pathways and metaCyc Metabolic Pathways were performed by PICRUSt 2 according to the standard procedures [19]. linear discriminate analysis effect size (LEfSe) was used to identify the bacterial taxa and KEGG pathways exhibiting differential abundances among the three groups.

### Measurement of rumen fermentation parameters

The rumen pH was immediately measured after collection using the Mettler Toledo Delta 320 pH meter (Mettler Toledo, Greifensee, Switzerland). The concentrations of VFAs including acetate, propionate, and butyrate in the rumen were determined by high-performance liquid chromatography (HPLC) using an LC-10A system (Tokyo, Japan). Specifically, a Shodex RSpak KC-811S-DVB gel C column (8.0 mm×30 cm) was applied under an oven temperature of 30°C with 3 mmol/L of HClO4 as the mobile phase at a flow rate of 1.0 mL/min. A total of 5-μL sample supernatant was injected for each measurement and data were collected using the SPD-M10AVP detector. Total VFA (TVFA) is defined as the sum of acetate, propionate, and butyrate concentrations. Ammonia nitrogen (NH_3_-N) concentration was determined by a spectrophotometer (Model 721/721-100; Shanghai, China) using ammonium chloride solution as standard.

### Determination of apparent digestibility of nutrients

Fresh fecal samples (250 g per animal), feed, and dried rice straw were collected daily during the last two weeks of the feeding experiment. Subsequently, all fecal samples were mixed, and 10% hydrochloric acid added to fix nitrogen. All samples were subjected to oven drying at 65°C for 72 h until constant weight. The nutrient contents in both the feed and the mixed feces were determined using AOAC methods (AOAC, 2001). Briefly, The dry matter (DM) and organic matter (OM) contents were determined according to the method described by Luo et al [6]. Crude protein (CP) was measured using the Kjeldahl nitrogen method and calculated as N×6.25. Crude fat (CF) was extracted with diethyl ether extraction using a Soxhlet apparatus. The acid detergent fiber (ADF) and neutral detergent fiber (NDF) contents were determined using the Van Soest methods [20]. Acid insoluble ash (AIA) was employed as an internal marker to calculate the apparent digestibility of nutrients as previously described by Luo et al [6]. The apparent digestibility of nutrients was calculated using the formula: apparent digestibility of a specific nutrient (%) = [1 − (nutrient content in feces × hydrochloric AIA content in feed) /(nutrient content in feed × hydrochloric AIA content in feces)] × 100.

### Statistical analysis

One-way analysis of variance analysis was performed to compare rumen fermentation parameters and apparent digestibility of nutrients using SPSS 19.00 software (SPSS Inc., Chicago, IL, USA). Multiple comparisons were conducted for identifying significant differences among the three groups. The results were expressed as mean values and the standard error of the mean. The significance thresholds were set at p≤0.01, p≤0.05, and 0.05<p≤0.10, respectively. The correlations between the concentrations of VFAs in the rumen and the abundances of OTUs were evaluated by the Spearman rank correlation analysis. The significance level was set at the false discover rate <0.05. The heatmap was plotted with the pheatmap package in R (v 3.3.1).

## RESULTS

### Effect of NA on the diversity of rumen bacterial composition

A total of 781,946 clean reads were obtained for all tested samples. To avoid the influence of sequencing depth on microbial composition, the sequencing depth for all tested samples was normalized to at least 15,803 tags (31,606 reads) per sample (the least number for individuals). The Good’s coverage based on the observed OTUs reached 98% (Table 2), and the rarefaction curves also achieved saturation (Supplementary Figure S1). These results indicated that the sequencing depth was sufficient.

**Figure 1:**
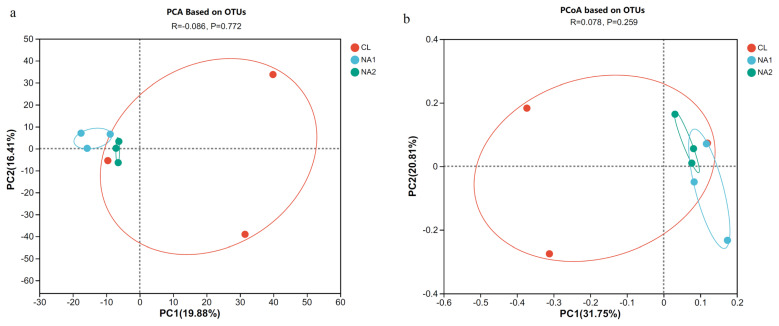
Effect of nicotinic acid (NA) on the beta-diversity of rumen bacterial composition in uncastrated bulls. (a) Principal component analysis (PCA) result based on OTUs. (b) Principal co-ordinates analysis (PCoA) result based on OTUs. OTUs, operational taxonomic units.

To investigate the effect of NA on rumen microbiota composition, the alpha-diversity and beta-diversity were compared among three groups. The results showed that the OTU number, diversity indices (Sobs and Ace), and richness index (Chao) in the NA1 group were significantly lower than those in CL and NA2 groups (p<0.05) (Table 2). In the PCA and PCoA, the samples from each of the CL, NA1, and NA2 groups were clustered into their corresponding group, except for one sample from the control group, which was close to the NA groups (Figure 1). The results also demonstrated that the inter-sample distances within each group were remarkably close, enabling their distinct separation from other groups, and suggested the significant effect of NA on rumen microbial compositions. The cattle from the two NA groups had a higher similarity of rumen microbial compositions than the cattle from the control group.

### Effect of dietary nicotinic acid on the composition and abundance of rumen bacterial taxa

**Figure 2:**
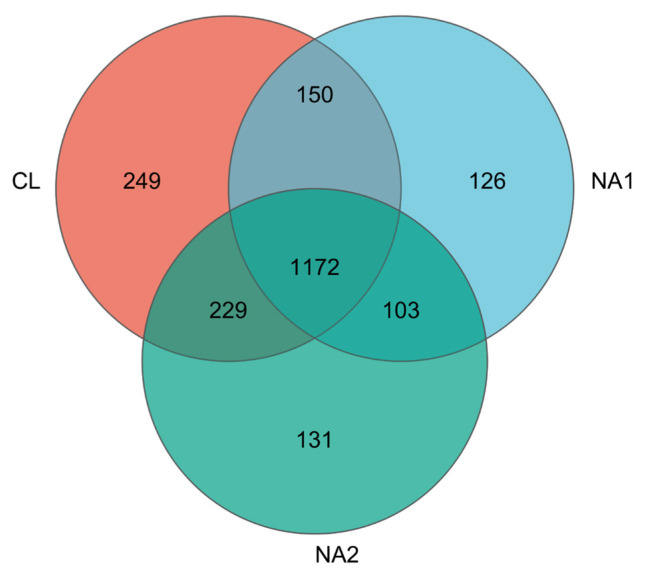
Venn diagram showing the numbers of operational taxonomic units (OTUs) shared among the three groups or unique to each group.

**Figure 3:**
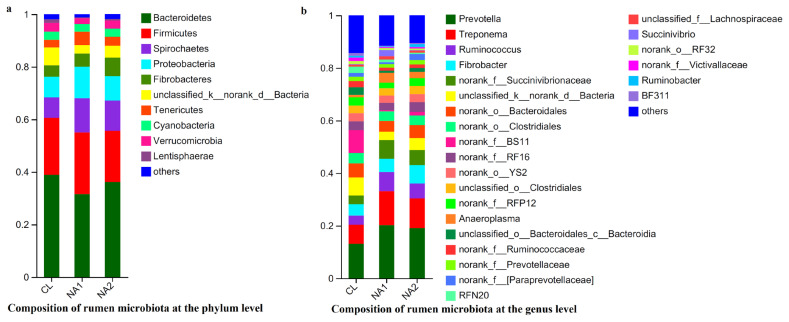
Histograms showing the compositions of rumen bacteria at the phylum (a) and genus level (b). The vertical axis indicates three experimental groups, and the horizontal axis shows the relative abundance of each taxonomy. The Histograms were visualized using R package.

Based on the 97% sequence identity, a total of 2,160 OTUs were obtained from all nine tested samples. Among them, 1,172 OTUs were shared in samples across all three groups. The number of OTUs that were specific to the CL, NA1, and NA2 groups was 249, 126, and 131, respectively (Figure 2). These 2,160 OTUs were annotated to a total of 21 bacterial phyla. After the phyla with low relative abundance were filtered, 10 bacterial phyla accounting for more than 99.0% of total abundance were left. The predominant phyla in bovine rumen were Bacteroidetes, Firmicutes, Spirochaetes, and Proteobacteria, which accounted for 31.89% to 41.22%, 18.78% to 22.24%, 8.20% to 13.75%, and 7.70% to 12.36% of relative abundances, respectively, followed by Fibrobacteres, Tenericutes, Cyanobacteria, Verrucomicrobia and Lentisphaerae (Figure 3a; Supplementary S2a). The relative abundance of Firmicutes was similar in all three experimental cattle groups (CL, 20.25%; NA1, 22.24%; and NA2, 18.78%) (Figure 3a; Supplementary Figure S2a). However, the NA1 (13.75%) and NA2 (12.02%) groups had a significantly higher relative abundance of Spirochaetes compared to the CL group (8.20%). Additionally, compared with the CL group, there was also a significant increase in the relative abundance of Tenericutes (CL vs NA1: 2.57% vs 4.93%) and Proteobacteria (CL vs NA1: 7.70% vs 12.36%) in the NA1 group as well (Figure 3a; Supplementary Figure S2a). However, the relative abundances of Bacteroidetes (CL vs NA1 vs NA2: 41.22% vs 31.89% vs 37.22%) and Lentisphaerae (CL vs NA1 vs NA2: 1.30% vs 0.34% vs 048%) were decreased in the NA1 and NA2 groups compared to the CL group. The relative abundances of other bacterial phyla were not significantly altered by the intervention of dietary NA. At the genus level, *Prevotella*, *Treponema*, *Ruminococcus*, and *Fibrobacter* were identified as the predominant genera in the rumen (Figure 3b; Supplementary Figure S2b). The relative abundances of *Prevotella*, *Treponema*, and *Ruminococcus* were higher in both NA1 and NA2 groups than that in the CL group. Conversely, the abundance of *Bacteroidia* was lower in both NA1 and NA2 groups than that in the CL group (Figure 3b; Supplementary Figure S2b).

**Figure 4:**
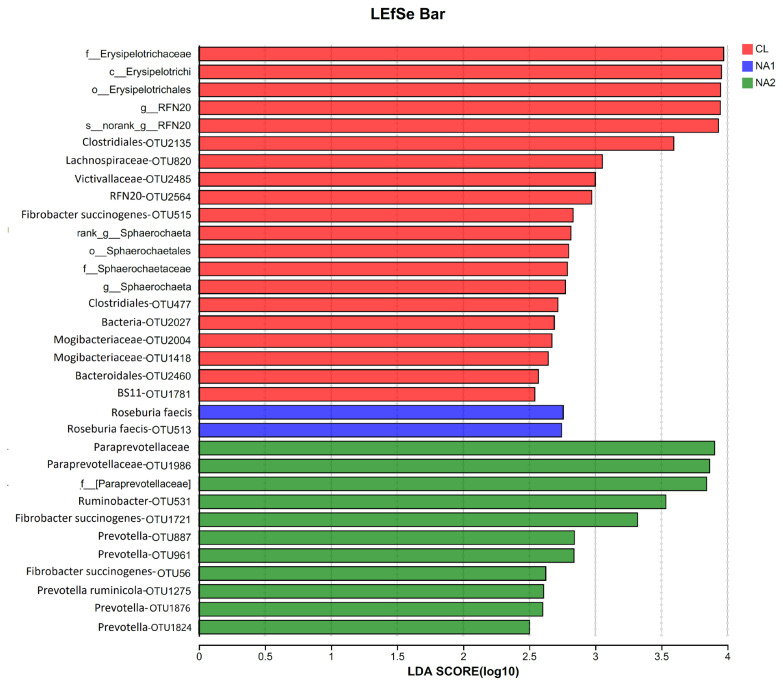
Rumen bacterial taxa showing differential abundances among the three experimental groups identified by LEfSe analysis. The *x*-axis shows the LDA scores of differential bacterial taxa, and the *y*-axis indicates the differential bacterial taxa. The taxa with LDA score >3 are shown. LEfSe, linear discriminate analysis effect size; LDA, linear discriminant analysis.

LEfSe analysis was further employed to identify the differential bacterial taxa among the three groups. At the significant threshold of linear discriminant analysis (LDA) ≥2 and p<0.05, a total of 78 bacterial taxa showed different enrichments among three experimental cattle groups (Figure 4; Supplementary Table S1). Most numbers of 47 bacterial taxa were enriched in the CL group. Among these 47 differential bacterial taxa, eight taxa belonged to Erysipelotrichi in which the taxa from Erysipelotrichi to the genus *RFN20*, and to *RFN20* OTUs showed enrichment in the CL group. A total of 15 bacterial taxa belonging to Clostridiales were also enriched in the CL group. Among them, five taxa were classified into Ruminococcaceae, three taxa into Mogibacteriaceae, and two taxa into Lachnospiraceae. Five taxa belonging to Sphaerochaetales (three taxa were annotated to *Sphaerochaeta*) and six taxa classified into Pedosphaerales were also enriched in the control group. A total of 11 bacterial taxa had higher abundances in the NA1 group (800 mg/kg NA), including two taxa belonging to *Roseburia faecis*. There were 20 taxa showing enrichments in the NA2 group, including seven taxa belonging to *Prevotella*, three taxa classified into Paraprevotellaceae, three taxa belonging to Bifidobacteriaceae, and two OTUs annotated to *Fibrobacter succinogenes* (LDA value >2.0 and p<0.05) (Figure 4; Supplementary Table S1).

### Effect of dietary nicotinic acid on the potential functional capacities of rumen microbiota

**Figure 5:**
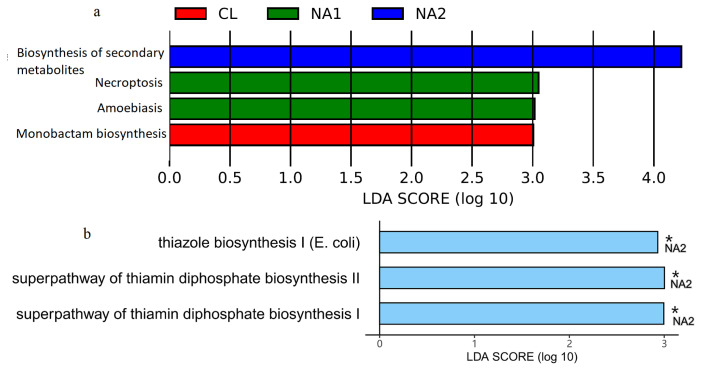
The differential function pathways identified by LEfSe analysis. Potential function pathways of rumen microbiome was predicted by PICRUSt 2. (a) The differential KEGG pathways. (b) The differential MetaCyc pathways. LEfSe, linear discriminate analysis effect size; KEGG, Kyoto encyclopedia of genes and genomes; LDA, linear discriminant analysis. The significant threshold was set at the LDA>2.0 and p<0.05.

Potential functional capacities of rumen microbiota were predicted by PICRUSt 2 with 16S rRNA marker gene sequences for all nine experimental samples based on KEGG pathways and metaCyc metabolic pathways. Metabolic pathways, biosynthesis of secondary metabolites, biosynthesis of amino acids, microbial metabolism in diverse environments, and carbon metabolism were the KEGG pathways whose abundances were ranked in the top five in all nine samples. Pentose phosphate pathway (non-oxidative branch), gondoate biosynthesis (anaerobic), adenosine ribonucleotides de novo biosynthesis, cis-vaccenate biosynthesis, and superpathway of pyrimidine nucleobases salvage were the top five metaCyc metabolic pathways according to their abundances in the tested samples. LefSe analysis was used to identify the differential pathways among the three groups. At the significance threshold of LDA>2.0 and p<0.05, the KEGG pathway of biosynthesis of secondary metabolites was enriched in the NA2 group, the pathways of Necroptosis and Amoebiasis had higher abundances in the NA1 group, and Monobactam biosynthesis showed the enrichment in the CL group (LDA> 3.0, p<0.05) (Figure 5a). For the metaCyc metabolic pathways, superpathway of thiamin diphosphate biosynthesis I, superpathway of thiamin diphosphate biosynthesis II, and thiazole biosynthesis I (*Escherichia coli*) were significantly enriched in the NA2 group (LDA value >2.0 and p<0.05). However, we did not identify any pathways enriched in the CL and NA1 groups (Figure 5b).

### Effect of dietary nicotinic acid on rumen fermentation parameters and apparent digestibility of nutrients

To investigate the impact of supplemental NA on rumen fermentation and digestion, the rumen fermentation parameters and apparent digestibility of nutrients were measured for all 27 experimental cattle. As shown in Table 3, NA supplementation showed certain effects on rumen fermentation in uncastrated bulls. The concentrations of acetate, TVFA, and the acetate/propionate ratio (A/P) in the rumen were significantly increased in both NA1 and NA2 groups (p<0.05). In addition, the concentration of butyrate was also significantly increased in the NA2 group (p<0.05). When comparing the proportion of individual VFA, it was observed that the proportion of acetate was significantly increased in both the NA1 group and NA2 groups. However, there was a significant decrease in the proportion of propionate in both NA treatment groups. However, dietary NA had no significant effects on rumen pH, NH_3_-N, propionate concentration, and butyrate proportion among the three groups (p>0.05).

As shown in Table 4, the apparent digestibility of ADF exhibited a significant increase in both NA1 and NA2 groups (p<0.01). Conversely, the apparent digestibility of DM and OM demonstrated a decrease in the NA2 group. No statistically significant differences were observed in the apparent digestibility of CP, CF, and NDF among all three groups (p> 0.05).

### The interplay patterns between rumen fermentation and bacterial community

**Figure 6:**
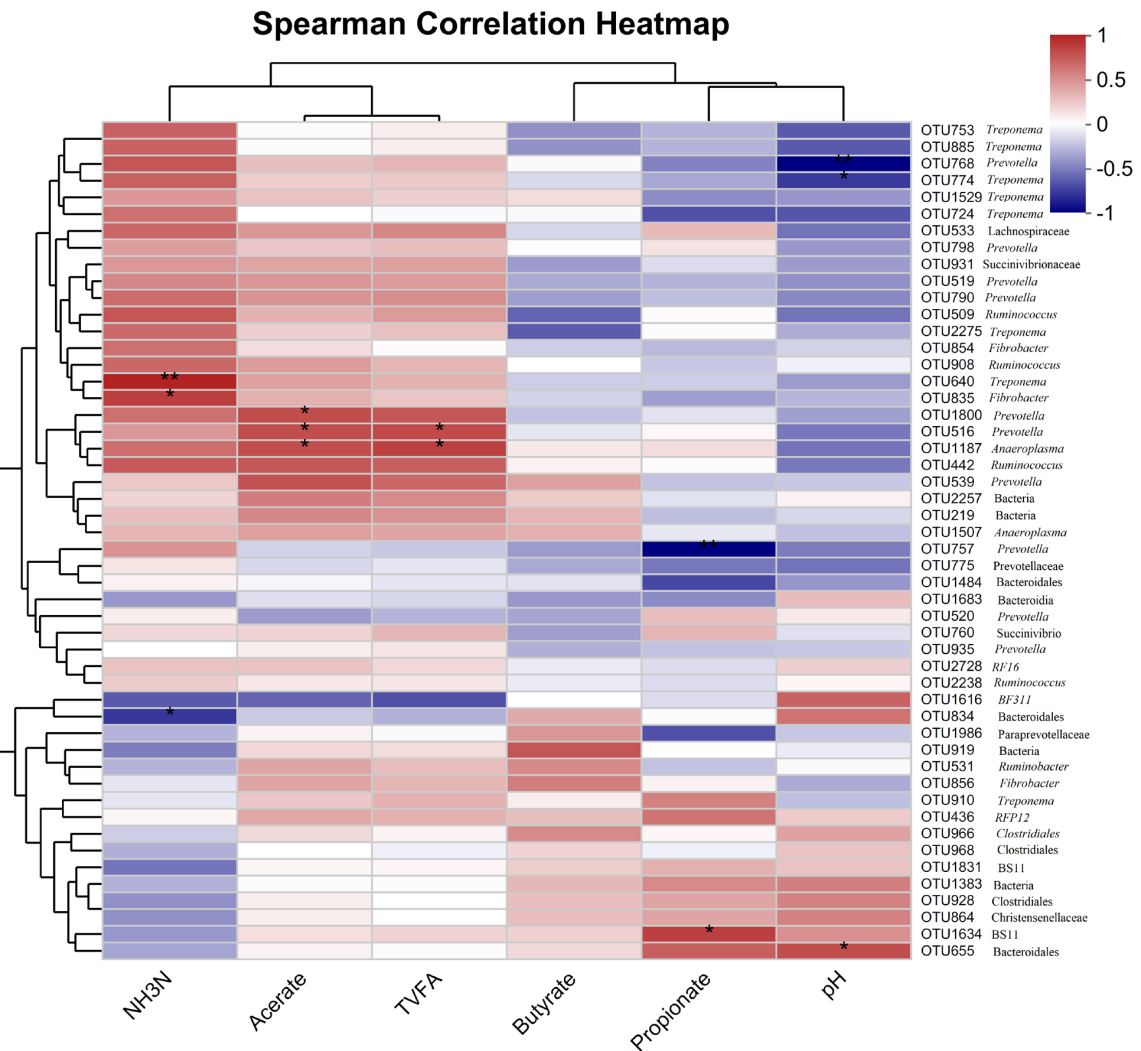
Heatmap of the correlations between the abundances of rumen bacterial OTUs, and the concentrations of rumen volatile fatty acids and pH values. The *x*-axis shows rumen volatile fatty acids and pH values, and the *y*-axis indicates OTUs (the top 50 OTUs in relative abundances) (right) and their annotations to bacterial taxa (Left). The correlation coefficient values (*r*) are shown with color gradients. The legend on the right is the color gradient of different *r* values. Orange color indicates positive correlation, and blue color indicates negative correlation. * Means 0.01<p≤0.05, and ** means 0.001<p≤0.01.

To evaluate the correlation between rumen fermentation parameters and the rumen microbial compositions, a correlation analysis was conducted between the concentrations of rumen VFAs and the abundances of 50 OTUs with high abundance in tested samples (Figure 6; Supplementary Table S2). l *r* l >0.5 and p<0.05 was set as the threshold of significant correlations, and l *r* l >0.5 was treated as the tendency of correlation. A total of 13 significant correlations and 35 tendencies were obtained (Figure 6; Supplementary Table S2). In details, acetate concentration exhibited a strong association with six OTUs (l *r* l >0.5), among which the OTU annotated to *BF311* displayed negative correlation with acetate concentration (*r* = −0.53), while the OTU442 annotated to *Ruminococcus* (*r* = 0.63), OTU539, OTU1800, and OTU516 to *Prevotella* (*r* = 0.65, 0.67, and 0.67, p<0.05), and the OTU1187 to *Anaeroplasma* (*r* = 0.67, p<0.05) showed a positive correlation with acetate concentration. Propionate concentrations in the rumen showed significant correlations with the abundances of seven OTUs (l *r* l >0.5), among them, the OTU1484 annotated to *Bacteroidales* (*r* = −0.63), OTU724 to *Treponema* (*r* = −0.60), OTU1986 to *Paraprevotellaceae* (*r* = −0.60), and OTU757 to *Prevotella* (*r* = −0.87, p<0.05) exhibited negative correlations with propionate levels of rumen fluid, while the OTU436 annotated to *RFP12* (*r* = 0.52), OTU655 to bacteria (*r* = 0.60), and OTU1634 to *BS11* (*r* = 0.72, p<0.05) displayed positive associations with propionate concentration. Butyrate concentrations in the rumen were negatively correlated with the OTU509 annotated to *Ruminococcus* (*r* = −0.53) and OTU2275 to *Treponema* (*r* = −0.56). However, the OTU919 (Bacteria) was positively associated with the concentration of butyrate. The concentration of TVFA exhibited a negative correlation with the OTU1616 annotated to *BF311* (*r* = −0.60). However, the OTUs annotated to *Ruminococcus* (*r* = 0.60), *Prevotella* (*r* = 0.68, p<0.05), and *Anaeroplasma* (*r* = 0.71, p<0.05) showed positive correlations with TVFA. The greatest number of 16 OTUs showed positive correlations with the NH_3_-N concentrations (*r*>0.50). These OTUs were annotated to *Prevotella*, *Ruminococcus*, *Fibrobacter* (*r* = 0.72, p<0.05), and *Treponema* (*r* = 0.83, p<0.05), while negative correlations were observed on the OTU1616 annotated to *BF311* (*r* = −0.53) and OTU834 to *Bacteroidales* (*r* = −0.68, p<0.05). Rumen pH exhibited a positive correlation with the OTU1616 annotated to *BF311* (*r* = −0.58). A total of five OTUs were negatively associated with rumen pH values. Among them, four OTUs belonged to *Treponema* (*r* = −0.57 to −0.68, p<0.05), and the other one OTU to *Prevotella* (*r* = 0.87, p<0.05).

## DISCUSSION

Previous studies have demonstrated that NA supplementation can modulate rumen fermentation patterns and the composition of rumen microbiota [7,9,12,13]. However, to our knowledge, there are few studies about the effect of NA on rumen microbiota in uncastrated bulls. In this study, we systematically investigated the effect of the supplementation of dietary NA on apparent digestibility of nutrients, rumen fermentation parameters, and rumen microbiome in uncastrated Xiangzhong black cattle. The study would benefit the regulation of rumen microbiota to improve feed efficiency by feed additives, such as NA.

VFAs produced by rumen microbial fermentation serve as a crucial indicator of rumen fermentation capacity and provide approximately 70% to 80% of energy for the host. The concentration of rumen TVFA exhibited an increase in both NA1 and NA2 groups. This finding was consistent with the previous reports by Samanta et al [21] and Ghosh et al [22], which suggested higher TVFA concentrations in cattle fed diets supplemented with NA. Acetate serves as a precursor for fat synthesis and promotes lipogenesis [3]. Butyrate can be metabolized into acetyl-CoA in the body to facilitate lactose and milk fat synthesis [3]. The present study revealed significant increases in acetate concentration and A/P ratio in both NA1 and NA2 groups. And an additional increase in butyrate concentration was observed in the NA2 group. These findings suggest that NA may have a positive impact on energy metabolism in the body and facilitate muscle fat deposition. Our previous studies has also proved that the beneficial effects of NA on intramuscular fat deposition and lipid metabolism in fattening steers [23].

As for the regulation of NA in apparent digestibility of nutrients, some studies have shown that dietary NA has a significant effect on apparent digestibility of nutrients [24,25]. Our findings also indicated that the supplementation of 800 mg/kg NA significantly enhanced the apparent digestibility of ADF but showed no effect on the digestibility of NDF. Furthermore, the supplementation of 1,200 mg/kg NA reduces the apparent nutrient digestibility of DM and OM. Luo et al [6] also found that dietary NA increased the apparent digestibility of ADF in beef cattle. However, this finding did not agree with the results in other studies [8–10,25]. These inconsistent results might be attributed to the variations in sex, age and breed of experimental cattle, and the diets provided to experimental animals.

The dominant phyla in uncastrated bulls were Bacteroideta, Firmicutes, Spirochaeta, and Proteobacteria. This result was consistent with the report in Chinese cattle by Luo et al [7]. Several previous studies have demonstrated the roles of NA in modulating microbial composition [7,9,21,22]. Here, we also revealed that dietary NA significantly changed the diversity of rumen microbiota in both alpha- and beta-diversity analyses, and the abundances of tens of bacterial taxa. Specially, the supplementation of 1,200 mg/kg NA in diets significantly increased the abundances of 20 bacterial taxa in the rumen. Previous studies indicated that NA exhibits potent antioxidant and anti-inflammatory properties and acts as a modulator of bacterial endotoxin production [26,27]. The intake of niacin (900 to 3,000 mg) could lead to a significant increase in the population of Bacteroidetes [28]. Interestingly, 12 out of these 20 bacterial taxa belonging to Bacteroidetes (Supplementary Table S1). By combining the results of VFA concentrations in the rumen, apparent digestibility of nutrients, and the composition and abundances of rumen microbiota, we inferred the possible mechanism of NA influencing apparent digestibility of nutrients and rumen fermentation through regulating rumen microbiota as follows.

First, dietary NA, especially the supplementation of 1,200 mg/kg NA, increased the abundances of bacterial taxa involved in the metabolisms of a wide range of proteins and polysaccharides. The relative abundance of *Prevotella* was significantly increased in both NA1 and NA2 groups, especially in the NA2 group in which seven OTUs belonging to *Prevotella* had significantly higher abundance (Figure 4; Supplementary Table S1). Interestingly, this observation was consistent with the finding in pigs, in which the gut microbiota of pigs fed with commercial formula diets (high protein and energy contents) had significantly high abundance of *Prevotella* [29]. *Prevotella*, a dominant genus of rumen microbiota, is a major proteolytic and amylolytic genus [30]. *Prevotella* is a very versatile microbe containing highly active proteolytic enzymes and hemicellulolytic enzymes [31] and capable of processing a wide range of proteins and polysaccharides to produce VFAs [32]. *Prevotella* can effectively degrade starch, pectin and xylan into acetate, propionate and succinate [12,33–35]. This was an exact explanation of the positive correlations between the abundances of *Prevotella* OTUs and the concentrations of acetate and TVFA in the rumen. These results indicated that *Prevotella* proliferation caused by NA play important roles in protein metabolism of high-concentrate and rumen fiber fermentation.

Three taxa belonging to *Bifidobacteriaceae* were enriched in the NA2 group (Figure 4; Supplementary Table S1). *Bifidobacteriaceae* genomes are comprised of a large number of enzymes involved in glucose metabolism and carbohydrate modification, including glycosylhydrolase, carbohydrate esterase, polysaccharidelyase, glycosyltransferase, and other carbohydrate active enzymes that participate in downstream metabolic processes [36]. These enzymes facilitate the fermentation of dietary carbohydrates into short-chain fatty acids such as acetate and propionate [36]. Two OTUs belonging to *Fibrobacter succinogenes* also had higher abundances in the NA2 groups compared to the control group. *Fibrobacter succinogenes* is a major contributor to cellulose digestion in the rumen of cattle [37]. It is capable of breaking down many sugars to produce formate, acetate, and succinate [38]. All these results were concordant with that the supplementation of 1,200 mg/kg NA elevated the concentrations of acetate, butyrate, and TVFA in the rumen. Three taxa classified into Paraprevotellaceae were also enriched in the NA2 group. A previous study also reported that the OTUs belonging to the families Bifidobacteriaceae, Paraprevotellaceae, and Prevotellaceae were associated with an increased feed efficiency in beef cattle [39]. In this study, the apparent digestibility of ADF was also improved significantly. Strangely, the apparent digestibility of NDF was not significantly changed by dietary NA. This might be due to different nutrient components showed different priorities by bacterial digestion and utilization. However, further experiments would need to be performed to confirm this inference.

Second, dietary NA decreased the abundances of bacterial taxa that are the most common fiber-degrading and VFAs-producing bacteria in uncastrated cattle fed with high-concentrate diet. For examples, most species of Clostridiales are saprophytic organisms that ferment plant polysaccharides to produce VFAs [40]. In this study, the abundances of 15 bacterial taxa belonging to Clostridiales including five Ruminococcaceae taxa and two Lachnospiraceae taxa were significantly decreased. Most members in Ruminococcaceae in the rumen are cellulolytic bacteria [41] that are capable of hydrolyzing cellulose and releasing esterified phenolic acids from plant cellulose [42], and play an important role in the production of butyrate and other VFAs [43]. The Lachnospiraceae are a family of obligately anaerobic and variably spore-forming bacteria that ferment diverse plant polysaccharides [11] to VFAs (butyrate and acetate) [40]. Dietary NA also decreased the abundances of five *Sphaerochaeta* taxa. *Sphaerochaeta* belongs to the family Spirochaetaceae and comprises anaerobic bacteria with fermentative metabolism. This genus contains large gene clusters characteristic of the order Clostridiales and encoding the proteins of metabolic pathways, including those of carbohydrate metabolism [44]. The significantly decreased abundances of these most common fiber-degrading and VFAs-producing bacteria in uncastrated cattle fed with 1,200 mg/kg NA should be the reason that can be used to explain why the apparent digestibility of DM and OM was lower in the NA2 group (1,200 mg/kg NA) than that in the control group. There was no significant difference in rumen pH among the three groups because all bacterial taxa up- and down-regulated by dietary NA belong to the VFAs-producing bacteria.

Third, dietary NA also decreased the abundances of bacterial taxa affecting host lipid and cholesterol metabolism. The bacterial taxa belonging to Erysipelotrichaceae was significantly enriched in the CL group (or a notable reduction in the relative abundance by dietary NA). A large number of studies have demonstrated the involvement of *Erysipelotrichaceae* in host lipid and cholesterol metabolism [45–49]. Notably, a positive correlation has been observed between the abundance of *Erysipelotrichchaceae* and liver fat content in choline deficient females [47]. Our previous study has also demonstrated that NA can reduce the carcass fat content of Xiangzhong black cattle, while increasing the muscle fat content [23]. Additionally, supplementary NA in diets significantly decreased serum concentrations of total cholesterol and high-density lipoprotein cholesterol (HDL-C), and increased serum concentrations of non-esterified fatty acids and glucose in uncastrated bulls [13]. Therefore, we hypothesized that NA may affect the body fat metabolism by altering rumen *Erysipelotrichaceae* abundance. To our knowledge, for the first time, our findings represent the effect of dietary NA on *Erysipelotrichaceae* abundance in the rumen.

The potential functional capacity of rumen microbial community was predicted using PICRUSt 2. Differential KEGG and metaCyc pathway analysis suggested that dietary NA strengthened the biosynthesis of secondary metabolites, such as thiamin diphosphate. However, only a few pathways showed differential abundances among the three experimental cattle groups. This should be due to the small sample size. Metagenomic sequencing analysis should be performed in the future study to further evaluate the effect of dietary NA on the functional capacity of rumen microbiota.

## CONCLUSION

In summary, dietary NA enhanced the digestibility of ADF, increase the concentrations of acetate and TVFA, as well as the ratio of A/P. Dietary NA significantly changed the diversity of rumen microbiota and the abundances of tens of bacterial taxa in the rumen. The relative abundances of bacterial taxa belonging to Erysipelotrichchaceae, Ruminococcaceae, Lachnospiraceae, and *Sphaerochaeta* were significantly decreased by dietary NA, while 1,200 mg/kg NA significantly increased the abundances of the taxa annotated to *Prevotella*, Paraprevotellaceae, and Bifidobacteriacea. Except Erysipelotrichchaceae taxa, all the taxa changed by NA are VFAs-production bacteria. We also found the correlations between the concentrations of VFAs and the abundances of bacterial taxa in the rumen. We suggested that dietary NA should influence rumen fermentation through regulating the rumen microbiota. The study provided the knowledge for improving rumen fermentation and nutrient digestibility by regulating rumen microbiota with feed additives.

## Figures and Tables

**Table 1:** Composition and nutrient levels of the basal concentrate diet (air-dry basis)

Items	Content (%)
Ingredients	
Corn	50.00
Cottonseed meal	23.00
Wheat bran	20.00
NaCl	1.00
NaHCO3	1.00
Mineral–vitamin premix^1)^	5.00
Total	100
Nutrient levels^2)^	
Dry matter	84.16
Crude protein	18.48
Crude fat	3.53
Acid detergent fibre	20.42
Neutral detergent fibre	38.33
Calcium	0.54
Total phosphorus	0.74

1) Mineral–vitamin premix (per kg): vitamin A, 250,000 IU; vitamin D_3_, 40,000 IU; vitamin E, 1,000 IU; Cu, 1 g; Fe, 5 g; Mn, 4 g; Zn, 3 g; Se, 10 mg; I,50 mg; Co, 10 mg, Mg, 50g.

2) Nutrient levels as calculated value.

**Table 2:** Comparison of the α-diversity of rumen bacterial composition among three experimental groups treated with different dosages of dietary nicotinic acid (n = 3 for each group)

Items	Supplemental levels of NA (mg/kg)^1)^			SEM	p-value

	CL	NA1	NA2		
OTU number	1,163.67^a^	952.67^b^	1,130^a^	27.32	0.04
Sobs	1,281.70^a^	1,046.30^b^	1,238.70^a^	26.21	0.04
Shannon	5.54	5.33	5.53	0.06	0.32
Simpson	0.02	0.02	0.01	0.003	0.49
Chao	1,586.30^a^	1,297.10^b^	1,531.10^a^	28.61	0.03
ACE	1,590.20^a^	1,308.20^b^	1,556.90^a^	25.09	0.02
Good’s coverage	98.00	98.00	98.00	0.0003	0.43

Statistical analyses were performed with Wilcoxon rank sum test among three groups.

NA, nicotinic acid; SEM, standard error of the mean value; OTU, operational taxonomic unit; Sobs, the observed species; ACE, abundance-based coverage estimator.

1) CL, control group (0 mg/kg NA in concentrate diet); NA1 group, 800 mg/kg NA in concentrate diet; NA2 group, 1,200 mg/kg NA in concentrate diet.

a,b Mean values in the same row with no common superscript differ significantly (p<0.05).

**Table 3:** Effect of dietary NA on rumen fermentation parameters in uncastrated bulls

Item	Values of measurements^1)^			SEM	p-value

	CL	NA1	NA2		
pH value	6.40	5.85	5.79	4.34	0.11
NH_3_-N (mg/100 mL)	23.09	22.95	15.36	3.13	0.58
Acetate (mmol/L)	51.47^a^	69.42^b^	73.82^b^	0.89	0.01
Propionate (mmol/L)	11.96	12.65	10.43	0.37	0.12
Butyrate (mmol/L)	6.51^a^	7.38^a^	9.14^b^	0.21	0.01
TVFA (mmol/L)	69.92^a^	89.44^b^	93.39^b^	1.21	0.01
Acetate/TVFA (%)	73.66^a^	77.61^b^	79.03^b^	0.34	0.01
Propionate/TVFA (%)	17.06^a^	14.14^b^	11.14^b^	0.26	0.01
Butyrate/TVFA (%)	9.28	8.25	9.83	0.28	0.15
A/P	4.33^a^	5.49^b^	7.13^c^	0.13	0.01

n = 3 cattle per group.

NA, nicotinic acid; SEM, standard error of the mean value; TVFA, total volatile fatty acid; A/P, acetate to propionate.

1) CL, control group (0 mg/kg NA in concentrate diet); NA1 group, 800 mg/kg NA in concentrate diet; NA2 group, 1,200 mg/kg NA in concentrate diet.

a,b Mean values in the same row with different superscripts represent significant difference (p<0.05).

**Table 4:** Effect of NA on the apparent nutrient digestibility in uncastrated bulls

Items	Values of apparent nutrient digestibility^1)^			SEM	p-value

	CL	NA1	NA2		
DM (%)	49.04^a^	50.61^a^	41.70^b^	1.10	0.01
OM (%)	51.50^a^	53.62^a^	44.99^b^	1.14	0.02
CP (%)	57.72	59.07	58.17	0.79	0.78
CF (%)	59.38	56.29	53.22	1.57	0.29
ADF (%)	41.92^a^	47.13^b^	46.33^b^	0.65	0.01
NDF (%)	47.50	44.08	40.77	1.35	0.17
DMI (kg/d)[13]	10.52	10.84	10.72	0.07	0.15

n = 7 cattle per group.

NA, nicotinic acid; SEM, standard error of the mean; DM, dry matter; OM, organic matter; CP, crude protein; CF, crude fat; ADF, acid detergent fibre; NDF, neutral detergent fibre; DMI, dry matter intake.

1) CL, control group (0 mg/kg NA in concentrate diet); NA1 group, 800 mg/kg NA in concentrate diet; NA2 group, 1,200 mg/kg NA in concentrate diet.

a,b The mean values in the same row with different superscript are different (p<0.01).

## SUPPLEMENTARY MATERIAL

**Supplementary Table S1**. The bacterial taxa enriched in each group by LefSe analysis at the threshold of LDA>2.0

**Supplementary Table S2**. The correlation coefficients between the relative abundance of OTUs and the values of rumen fermentation parameters

**Supplementary Figure S1**. Rarefaction curves of observed OTU numbers following the sequencing depth.

**Supplementary Figure S2**. Circos diagram showing the relative abundances of bacterial taxa in samples of each group at the phylum (a) and genus level (b).

## References

[1] Flachowsky G (1993). Niacin in dairy and beef cattle nutrition. Archiv fur Tierernahrung.

[2] Chilliard Y (1993). Dietary fat and adipose tissue metabolism in ruminants, pigs, and rodents: a review. J Dairy Sci.

[3] Kristensen NB, Harmon DL (2004). Splanchnic metabolism of volatile fatty acids absorbed from the washed reticulorumen of steers. J Anim Sci.

[4] Pescara JB, Pires JAA, Grummer RR (2010). Antilipolytic and lipolytic effects of administering free or ruminally protected nicotinic acid to feed-restricted Holstein cows. J Dairy Sci.

[5] Titgemeyer EC, Spivey KS, Mamedova LK, Bradford BJ (2011). Effects of pharmacological amounts of nicotinic acid on lipolysis and feed intake in cattle. Int J Dairy Sci.

[6] Luo D, Gao YF, Lu YY (2019). Niacin supplementation improves growth performance and nutrient utilisation in Chinese Jinjiang cattle. Italian J Anim Sci.

[7] Luo D, Gao Y, Lu Y (2017). Niacin alters the ruminal microbial composition of cattle under high-concentrate condition. Anim Nutr.

[8] Doreau M, Ottou JF (1996). Influence of niacin supplementation on in vivo digestibility and ruminal digestion in dairy cows. J Dairy Sci.

[9] Horner JL, Coppock CE, Moya JR (1988). Effects of niacin and whole cottonseed on ruminal fermentation, protein degradability, and nutrient digestibility. J Dairy Sci.

[10] Erickson PS, Trusk AM, Murphy MR (1990). Effects of niacin source on epinephrine stimulation of plasma nonesterified fatty acid and glucose concentrations, on diet digestibility and on rumen protozoal numbers in lactating dairy cows. J Nutr.

[11] Shah AM, Wang Z, Ma J (2023). Effects of uni and bilateral castration on growth performance and lipid metabolism in yellow cattle. Anim Biotechnol.

[12] Cotta MA (1992). Interaction of ruminal bacteria in the production and utilization of maltooligosaccharides from starch. Appl Environ Microbiol.

[13] Yang ZQ, Bao LB, Zhao XH (2015). Effects of nicotinic acid on growth performance, meat quality and serum biochemical parameters of uncastrated xiangzhong bulls. Chinese J Anim Nutr.

[14] Parada AE, Needham DM, Fuhrman JA (2016). Every base matters: assessing small subunit rRNA primers for marine microbiomes with mock communities, time series and global field samples. Environ Microbiol.

[15] Magoc T, Salzberg SL (2011). FLASH: fast length adjustment of short reads to improve genome assemblies. Bioinformatics.

[16] Edgar R (2018). Taxonomy annotation and guide tree errors in 16S rRNA databases. PeerJ.

[17] Schloss PD (2020). Reintroducing mothur: 10 years later. Appl Environ Microbiol.

[18] Werner JJ, Zhou D, Caporaso JG, Knight R, Angenent LT (2012). Comparison of Illumina paired-end and single-direction sequencing for microbial 16S rRNA gene amplicon surveys. ISME J.

[19] Douglas GM, Maffei VJ, Zaneveld JR (2020). PICRUSt2 for prediction of metagenome functions. Nat Biotechnol.

[20] Van Soest PJ, Robertson JB, Lewis BA (1991). Methods for dietary fiber, neutral detergent fiber, and nonstarch polysaccharides in relation to animal nutrition. J Dairy Sci.

[21] Samanta AK, Kewalramani N, Kaur H (2000). Effect of niacin supplementation on VFA production and microbial protein synthesis in cattle. Indian J Dairy Sci.

[22] Ghosh NR, Kewalramani N, Kaur H (2003). Comparative efficacy of niacin vs nicotinamide on rumen fermentation in buffaloes fed straw hased diets. Buffalo J.

[23] Yang ZQ, Bao LB, Zhao XH (2016). Nicotinic acid supplementation in diet favored intramuscular fat deposition and lipid metabolism in finishing steers. Exp Biol Med (Maywood).

[24] Harmon BG, Becker DE, Jensen AH, Baker DH (1969). Nicotinic acid--tryptophan relationship in the nutrition of the weanling pig. J Anim Sci.

[25] Aschemann M, Lebzien P, Huther L, Döll S, Südekum KH, Dänicke S (2012). Effect of niacin supplementation on digestibility, nitrogen utilisation and milk and blood variables in lactating dairy cows fed a diet with a negative rumen nitrogen balance. Arch Anim Nutr.

[26] Uebanso T, Shimohata T, Mawatari K, Takahashi A (2020). Functional roles of B-vitamins in the gut and gut microbiome. Mol Nutr Food Res.

[27] Zhong W, Li Q, Zhang W, Sun Q, Sun X, Zhou Z (2015). Modulation of intestinal barrier and bacterial endotoxin production contributes to the beneficial effect of nicotinic acid on alcohol-induced endotoxemia and hepatic inflammation in rats. Biomolecules.

[28] Fangmann D, Theismann E, Türk K (2018). Targeted Microbiome intervention by microencapsulated delayed-release niacin beneficially affects insulin sensitivity in humans. Diabetes Care.

[29] Chen C, Fang S, Wei H (2021). Prevotella copri increases fat accumulation in pigs fed with formula diets. Microbiome.

[30] Huws SA, Kim EJ, Lee MRF (2011). As yet uncultured bacteria phylogenetically classified as Prevotella, Lachnospiraceae incertae sedis and unclassified Bacteroidales, Clostridiales and Ruminococcaceae may play a predominant role in ruminal biohydrogenation. Environ Microbiol.

[31] Kabel MA, Yeoman CJ, Han Y (2011). Biochemical characterization and relative expression levels of multiple carbohydrate esterases of the xylanolytic rumen bacterium Prevotella ruminicola 23 grown on an ester-enriched substrate. Appl Environ Microbiol.

[32] Betancur-Murillo CL, Aguilar-Marin SB, Jovel J (2022). Prevotella: A key player in ruminal metabolism. Microorganisms.

[33] Gardner RG, Wells JE, Russell JB (1995). The cellular location of Prevotella ruminicola beta-1,4-D-endoglucanase and its occurrence in other strains of ruminal bacteria. Appl Environ Microbiol.

[34] Krause DO, Denman SE, Mackie RI (2003). Opportunities to improve fiber degradation in the rumen: microbiology, ecology, and genomics. FEMS Microbiol Rev.

[35] Jacobs DM, Gaudier E, van Duynhoven J (2009). Non-digestible food ingredients, colonic microbiota and the impact on gut health and immunity: a role for metabolomics. Curr Drug Metab.

[36] Milani C, Turroni F, Duranti S (2016). Genomics of the genus bifidobacterium reveals species-specific adaptation to the glycan-rich gut environment. Appl Environ Microbiol.

[37] Burnet MC, Dohnalkova AC, Neumann AP (2015). Evaluating models of cellulose degradation by fibrobacter succinogenes S85. PLoS One.

[38] Simunek J, Killer J, Sechovcova H (2018). Characterization of a xylanolytic bacterial strain C10 isolated from the rumen of a red deer (Cervus elaphus) closely related of the recently described species Actinomyces succiniciruminis, A. glycerinitolerans, and A. ruminicola. Folia Microbiol (Praha).

[39] Paz HA, Hales KE, Wells JE (2018). Rumen bacterial community structure impacts feed efficiency in beef cattle. J Anim Sci.

[40] Boutard M, Cerisy T, Nogue PY (2014). Functional diversity of carbohydrate-active enzymes enabling a bacterium to ferment plant biomass. PLoS Genet.

[41] Aurilia V, Martin JC, McCrae SI, Scott KP, Rincon MT, Flint HJ (2000). Three multidomain esterases from the cellulolytic rumen anaerobe Ruminococcus flavefaciens 17 that carry divergent dockerin sequences. Microbiology.

[42] Giraud I, Besle J, Fonty G (1997). Hydrolysis and degradation of esterified phenolic acids from the maize cell wall by rumen microbial species. Reprod Nutr Dev.

[43] Xie J, Li LF, Dai TY (2022). Short-chain fatty acids produced by ruminococcaceae mediate alpha-linolenic acid promote intestinal stem cells proliferation. Mol Nutr Food Res.

[44] Ritalahti KM, Justicia-Leon SD, Cusick KD (2012). Sphaerochaeta globosa gen. nov., sp. nov. and Sphaerochaeta pleomorpha sp. nov., free-living, spherical spirochaetes. Int J Syst Evol Microbiol.

[45] Kaakoush NO (2015). Insights into the role of erysipelotrichaceae in the human host. Front Cell Infection Microbiol.

[46] Fleissner CK, Huebel N, Abd El-Bary MM, Loh G, Klaus S, Blaut M (2010). Absence of intestinal microbiota does not protect mice from diet-induced obesity. Br J Nutr.

[47] Spencer MD, Hamp TJ, Reid RW, Fischer LM, Zeisel SH, Fodor AA (2011). Association between composition of the human gastrointestinal microbiome and development of fatty liver with choline deficiency. Gastroenterology.

[48] Turnbaugh PJ, Backhed F, Fulton L, Gordon JI (2008). Diet-induced obesity is linked to marked but reversible alterations in the mouse distal gut microbiome. Cell Host Microbe.

[49] Martinez I, Wallace G, Zhang C (2009). Diet-induced metabolic improvements in a hamster model of hypercholesterolemia are strongly linked to alterations of the gut microbiota. Appl Environ Microbiol.

