# Supplementary material for: Nicotinic acid changes rumen fermentation and apparent nutrient digestibility by regulating rumen microbiota in Xiangzhong black cattle
Source: Anim Biosci. 2023 Oct 31;37(2):240–52. doi: 10.5713/ab.23.0149 (PMC10766483; doi:10.5713/ab.23.0149)
Supplement: Supplementary file 1 [file ab-23-0149-Supplementary-Table-1.pdf]

Table S1. The bacterial taxa enriched in each group by LeFSe analysis at the threshold of LDA>2.0

Species name	group	Mean	LDA value	P value
Erysipelotrichales	CL	4.393	4.007	0.039
Erysipelotrichaceae.g__RFN20	CL	4.379	3.983	0.039
Erysipelotrichi	CL	4.393	3.975	0.039
Erysipelotrichaceae	CL	4.393	3.953	0.039
Erysipelotrichaceae.g__RFN20.s__norank_g__RFN20	CL	4.379	3.946	0.039
Clostridiales.OTU2135	CL	2.102	3.202	0.018
Lachnospiraceae.OTU820	CL	3.400	3.034	0.027
Mogibacteriaceae.OTU1418	CL	1.801	2.976	0.018
Erysipelotrichaceae.g__RFN20.OTU2564	CL	3.341	2.967	0.039
Victivallaceae.OTU2485	CL	3.311	2.955	0.034
Mogibacteriaceae.OTU2004	CL	2.102	2.921	0.018
Bacteroidales.OTU2460	CL	2.023	2.872	0.021
Fibrobacter succinogenes.OTU515	CL	3.193	2.826	0.047
Sphaerochaetaceae	CL	3.254	2.821	0.031
Sphaerochaetales	CL	3.254	2.795	0.031
Sphaerochaeta	CL	3.254	2.781	0.031
Sphaerochaeta	CL	3.254	2.771	0.031
Clostridiales.OTU477	CL	3.102	2.746	0.026
d__Bacteria.OTU2027	CL	2.977	2.741	0.034
d__Bacteria.OTU2145	CL	2.438	2.669	0.021
BS11.OTU1781	CL	2.892	2.543	0.048
Prevotellaceae.OTU1716	CL	2.787	2.525	0.031
RFN20.OTU1427	CL	2.739	2.470	0.046
YS2.OTU1570	CL	2.227	2.463	0.034
Victivallaceae.OTU2122	CL	1.926	2.371	0.021
R4-41B	CL	2.579	2.341	0.042
RFN20.OTU335	CL	2.625	2.336	0.050
R4-41B.OTU1745	CL	2.579	2.334	0.042
[Pedosphaerales]	CL	2.579	2.331	0.042
[Pedosphaerae]	CL	2.579	2.329	0.042
norank_f__R4-41B	CL	2.579	2.320	0.042
R4-41B	CL	2.579	2.312	0.042
Lachnospiraceae.OTU1916	CL	2.278	2.312	0.034
Clostridiales.OTU1885	CL	2.102	2.303	0.022
Ruminococcaceae.OTU1806	CL	2.555	2.287	0.046
[Mogibacteriaceae].OTU1181	CL	2.102	2.256	0.046
Ruminococcaceae.OTU2600	CL	2.102	2.242	0.046
Ruminococcaceae.OTU281	CL	2.169	2.220	0.048
Ruminococcaceae.OTU1688	CL	2.500	2.202	0.047
Clostridiales.OTU948	CL	2.403	2.202	0.045
Sphaerochaeta.OTU2036	CL	2.366	2.194	0.030
Anaeroplasma.OTU229	CL	2.403	2.192	0.046
BF311.OTU2681	CL	2.470	2.192	0.046
Clostridiales.OTU2639	CL	2.366	2.183	0.050
YS2.OTU1852	CL	2.227	2.181	0.034
Ruminococcaceae.OTU2134	CL	2.227	2.178	0.046
Bacteria.OTU2592	CL	2.227	2.081	0.046
Roseburia faecis	NA1	3.014	2.727	0.030
Roseburia faecis.OTU513	NA1	3.014	2.725	0.030
Clostridiales.OTU974	NA1	2.816	2.631	0.025
GMD14H09.OTU1221	NA1	2.816	2.497	0.050
Prevotella.OTU1891	NA1	2.500	2.423	0.048
Clostridiales.OTU2426	NA1	2.528	2.373	0.045
Clostridiales.OTU608	NA1	2.958	2.359	0.050
RF39.OTU189	NA1	2.603	2.330	0.041

Shuttleworthia.OTU548	NA1	2.366	2.229	0.032
Coriobacteriaceae.OTU2660	NA1	2.169	2.176	0.046
Lachnospiraceae.OTU1340	NA1	2.102	2.102	0.046
[Paraprevotellaceae].OTU1986	NA2	4.258	3.893	0.039
[Paraprevotellaceae]	NA2	4.366	3.885	0.039
norank_f__[Paraprevotellaceae]	NA2	4.366	3.854	0.039
Ruminobacter.OTU531	NA2	3.915	3.510	0.039
Fibrobacter succinogenes.OTU1721	NA2	3.636	3.291	0.027
Prevotella.OTU961	NA2	3.254	2.843	0.032
Prevotella.OTU887	NA2	2.102	2.650	0.018
Fibrobacter succinogenes.OTU56	NA2	2.904	2.633	0.035
Prevotella ruminicola.OTU1275	NA2	3.040	2.617	0.048
Prevotella.OTU1876	NA2	2.843	2.485	0.045
Clostridiales.OTU1115	NA2	1.926	2.450	0.021
Bacteroidales.OTU1515	NA2	2.756	2.408	0.048
Prevotella.OTU2241	NA2	2.686	2.379	0.045
Prevotella.OTU2283	NA2	2.470	2.330	0.041
Bacteroidia.OTU1606	NA2	2.470	2.238	0.035
Prevotella.OTU1824	NA2	2.366	2.214	0.034
YS2.OTU895	NA2	2.169	2.125	0.046
Bifidobacteriaceae	NA2	2.366	2.099	0.046
Bifidobacteriaceae.OTU2224	NA2	2.366	2.088	0.046
norank_f__Bifidobacteriaceae	NA2	2.366	2.073	0.046
