# Supplementary material for: Nicotinic acid changes rumen fermentation and apparent nutrient digestibility by regulating rumen microbiota in Xiangzhong black cattle
Source: Anim Biosci. 2023 Oct 31;37(2):240–52. doi: 10.5713/ab.23.0149 (PMC10766483; doi:10.5713/ab.23.0149)
Supplement: Supplementary file 2 [file ab-23-0149-Supplementary-Table-2.pdf]

**Table S2. The correlation coefficients between the relative abundance of OTUs and the values of rumen fermentation parameters**

| OTUs | genus | Acerate | Propionate | Butyrate | TVFA | $NH_3N$ | pH |
|---|---|---|---|---|---|---|---|
| OTU1616 | BF311 | **-0.533** | -0.133 | -0.017 | **-0.600** | **-0.567** | **0.583** |
| OTU520 | Prevotella | -0.350 | 0.233 | -0.317 | -0.267 | 0.050 | 0.067 |
| OTU834 | Bacteria | -0.192 | -0.025 | 0.310 | -0.276 | **-0.686** | **0.527** |
| OTU757 | Prevotella | -0.167 | **-0.867** | -0.350 | -0.200 | 0.400 | -0.450 |
| OTU775 | Prevotellaceae | -0.150 | -0.467 | -0.300 | -0.100 | 0.083 | -0.483 |
| OTU1683 | Bacteroidia | -0.126 | -0.402 | -0.360 | -0.151 | -0.360 | 0.243 |
| OTU1484 | Bacteroidales | -0.050 | **-0.633** | -0.117 | -0.100 | 0.033 | -0.367 |
| OTU1383 | Bacteria | -0.025 | 0.435 | 0.259 | 0.000 | -0.276 | 0.477 |
| OTU724 | Treponema | -0.017 | **-0.600** | -0.033 | -0.033 | **0.533** | **-0.583** |
| OTU968 | Clostridiales | -0.017 | -0.067 | 0.150 | -0.067 | -0.283 | 0.217 |
| OTU753 | Treponema | 0.000 | -0.267 | -0.383 | 0.050 | **0.583** | **-0.567** |
| OTU885 | Treponema | 0.000 | -0.267 | -0.383 | 0.050 | **0.583** | **-0.567** |
| OTU1831 | BS11 | 0.017 | 0.289 | 0.175 | 0.017 | -0.481 | 0.210 |
| OTU1986 | Paraprevotellaceae | 0.033 | **-0.600** | 0.383 | -0.033 | -0.267 | -0.200 |
| OTU655 | Bacteria | 0.033 | **0.600** | 0.133 | 0.000 | -0.317 | **0.667** |
| OTU935 | Prevotella | 0.050 | -0.217 | -0.283 | 0.083 | -0.017 | -0.200 |
| OTU928 | Clostridiales | 0.050 | 0.333 | 0.233 | -0.017 | -0.383 | 0.467 |
| OTU864 | Christensenellaceae | 0.050 | 0.333 | 0.233 | -0.017 | -0.383 | 0.467 |
| OTU2238 | Ruminococcus | 0.067 | -0.133 | -0.083 | 0.083 | 0.183 | 0.017 |
| OTU1634 | BS11 | 0.109 | **0.720** | 0.159 | 0.151 | -0.360 | 0.410 |
| OTU854 | Fibrobacter | 0.117 | -0.250 | -0.183 | 0.000 | **0.533** | -0.167 |
| OTU919 | Bacteria | 0.133 | -0.017 | **0.633** | 0.117 | -0.450 | -0.083 |
| OTU966 | Clostridiales | 0.133 | 0.017 | 0.433 | 0.033 | -0.183 | 0.350 |
| OTU760 | Succinivibrio | 0.153 | 0.271 | -0.339 | 0.271 | 0.136 | -0.119 |
| OTU2275 | Treponema | 0.168 | -0.025 | **-0.563** | 0.227 | **0.555** | -0.294 |
| OTU774 | Treponema | 0.176 | -0.310 | -0.142 | 0.192 | **0.594** | **-0.678** |
| OTU910 | Treponema | 0.200 | 0.467 | 0.050 | 0.283 | -0.100 | -0.233 |
| OTU798 | Lachnospiraceae | 0.201 | 0.092 | -0.025 | 0.234 | 0.360 | -0.360 |
| OTU1529 | Treponema | 0.217 | -0.400 | 0.117 | 0.167 | 0.383 | -0.367 |
| OTU2728 | RF16 | 0.217 | -0.133 | -0.083 | 0.133 | 0.217 | 0.167 |
| OTU768 | Prevotella | 0.233 | -0.433 | -0.033 | 0.267 | **0.633** | **-0.867** |
| OTU509 | Ruminococcus | 0.283 | 0.000 | **-0.533** | 0.367 | **0.633** | -0.483 |
| OTU835 | Fibrobacter | 0.286 | -0.336 | -0.168 | 0.202 | **0.723** | -0.261 |
| OTU436 | RFP12 | 0.317 | **0.517** | 0.233 | 0.283 | 0.017 | 0.183 |
| OTU931 | Succinivibrionaceae | 0.333 | -0.133 | -0.350 | 0.350 | 0.383 | -0.350 |
| OTU531 | Ruminobacter | 0.333 | -0.217 | 0.433 | 0.233 | -0.267 | -0.033 |
| OTU856 | Fibrobacter | 0.333 | 0.033 | 0.483 | 0.267 | -0.100 | -0.300 |
| OTU640 | Treponema | 0.350 | -0.183 | -0.183 | 0.283 | **0.833** | -0.350 |
| OTU1507 | Anaeroplasma | 0.351 | -0.092 | 0.293 | 0.335 | 0.268 | -0.226 |
| OTU908 | Ruminococcus | 0.367 | -0.200 | -0.017 | 0.267 | **0.567** | -0.067 |
| OTU533 | Prevotella | 0.383 | 0.250 | -0.150 | 0.450 | **0.567** | -0.483 |
| OTU519 | Prevotella | 0.383 | -0.267 | -0.300 | 0.367 | 0.450 | -0.383 |
| OTU790 | Prevotella | 0.400 | -0.233 | -0.333 | 0.417 | **0.550** | -0.417 |
| OTU219 | Bacteria | 0.450 | -0.233 | 0.267 | 0.400 | 0.233 | -0.150 |
| OTU2257 | Bacteria | 0.483 | -0.117 | 0.183 | 0.433 | 0.150 | 0.033 |
| OTU442 | Ruminococcus | **0.633** | 0.000 | 0.033 | **0.600** | **0.617** | -0.467 |
| OTU539 | Prevotella | **0.650** | -0.217 | 0.350 | **0.567** | 0.200 | -0.200 |

| | | | | | | | |
|---|---|---|---|---|---|---|---|
| OTU1800 | Prevotella | **0.667** | -0.117 | -0.217 | **0.633** | **0.533** | -0.333 |
| OTU516 | Prevotella | **0.667** | 0.017 | -0.100 | **0.683** | 0.383 | -0.467 |
| OTU1187 | Anaeroplasma | **0.667** | 0.117 | 0.067 | **0.717** | **0.550** | -0.483 |
