# Supplementary figures and images for: Nicotinic acid changes rumen fermentation and apparent nutrient digestibility by regulating rumen microbiota in Xiangzhong black cattle
Source: Anim Biosci. 2023 Oct 31;37(2):240–52. doi: 10.5713/ab.23.0149 (PMC10766483; doi:10.5713/ab.23.0149)

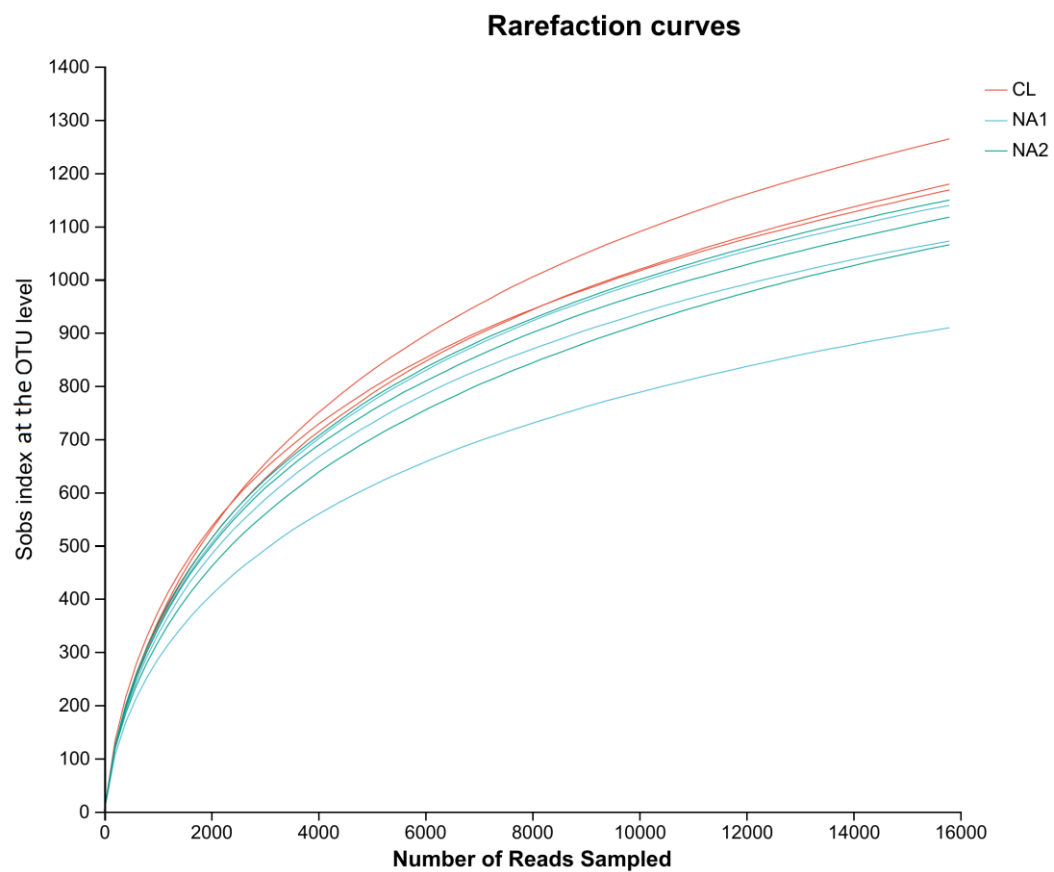

**Figure S1.** Rarefaction curves of observed OTU numbers following the sequencing depth.

Supplement: Supplementary file 3 [file ab-23-0149-Supplementary-Fig-1.pdf]
