# Supplementary material for: Nicotinic acid changes rumen fermentation and apparent nutrient digestibility by regulating rumen microbiota in Xiangzhong black cattle
Source: Anim Biosci. 2023 Oct 31;37(2):240–52. doi: 10.5713/ab.23.0149 (PMC10766483; doi:10.5713/ab.23.0149)
Supplement: Supplementary file 4 [file ab-23-0149-Supplementary-Fig-2.pdf]

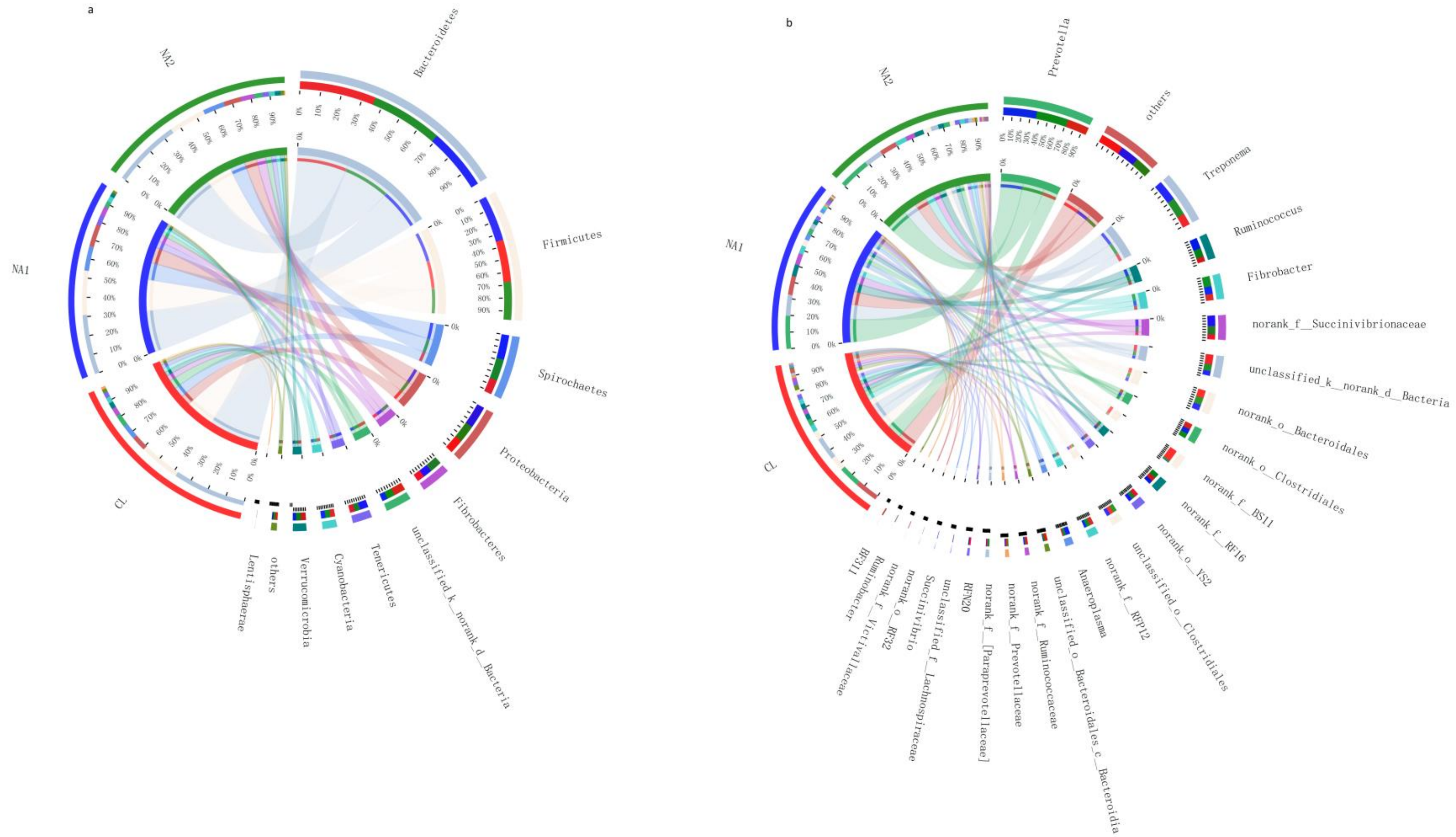

Figure S2. Circos diagram showing the relative abundances of bacterial taxa in samples of each group at the phylum (a) and genus level (b).
